# Development of a data classification system for preterm birth cohort studies: the RECAP Preterm project

**DOI:** 10.1186/s12874-021-01494-5

**Published:** 2022-01-07

**Authors:** Deborah Bamber, Helen E. Collins, Charlotte Powell, Gonçalo Campos Gonçalves, Samantha Johnson, Bradley Manktelow, José Pedro Ornelas, João Correia Lopes, Artur Rocha, Elizabeth S. Draper

**Affiliations:** 1 Department of Health Sciences, University of Leicester, Leicester, UK; 2 INESC TEC - Institute for Systems and Computer Engineering, Technology and Science, Porto, Portugal; 3 Faculdade de Engenharia da Universidade do Porto, Porto, Portugal

**Keywords:** RECAP preterm, Classification system, Metadata, Data harmonisation, Very preterm birth, Preterm birth cohort studies

## Abstract

**Background:**

The small sample sizes available within many very preterm (VPT) longitudinal birth cohort studies mean that it is often necessary to combine and harmonise data from individual studies to increase statistical power, especially for studying rare outcomes. Curating and mapping data is a vital first step in the process of data harmonisation. To facilitate data mapping and harmonisation across VPT birth cohort studies, we developed a custom classification system as part of the Research on European Children and Adults born Preterm (RECAP Preterm) project in order to increase the scope and generalisability of research and the evaluation of outcomes across the lifespan for individuals born VPT.

**Methods:**

The multidisciplinary consortium of expert clinicians and researchers who made up the RECAP Preterm project participated in a four-phase consultation process via email questionnaire to develop a topic-specific classification system. Descriptive analyses were calculated after each questionnaire round to provide pre- and post- ratings to assess levels of agreement with the classification system as it developed. Amendments and refinements were made to the classification system after each round.

**Results:**

Expert input from 23 clinicians and researchers from the RECAP Preterm project aided development of the classification system’s topic content, refining it from 10 modules, 48 themes and 197 domains to 14 modules, 93 themes and 345 domains. Supplementary classifications for target, source, mode and instrument were also developed to capture additional variable-level information. Over 22,000 individual data variables relating to VPT birth outcomes have been mapped to the classification system to date to facilitate data harmonisation. This will continue to increase as retrospective data items are mapped and harmonised variables are created.

**Conclusions:**

This bespoke preterm birth classification system is a fundamental component of the RECAP Preterm project’s web-based interactive platform. It is freely available for use worldwide by those interested in research into the long term impact of VPT birth. It can also be used to inform the development of future cohort studies.

**Supplementary Information:**

The online version contains supplementary material available at 10.1186/s12874-021-01494-5.

## Key messages

- A novel classification system was developed to facilitate harmonisation of data collected both retrospectively and prospectively from preterm birth cohort studies.
- The classification system was developed in consultation with clinicians and researchers experienced in studying the long term impact of preterm birth.
- The classification system will continue to develop as harmonised variables are created and as new cohort data collections join the RECAP Preterm platform.
- The classification system is freely available for use at https://platform.recap-preterm.eu/

## Background

Each year in Europe there are approximately 50,000 live births at very preterm gestations (VPT; < 32 weeks’ gestation). Although these only account for around 1% of all births across Europe, those born VPT account for up to half of all infant deaths [1]. Advances in obstetric and neonatal medicine have led to an increase in survival rates for these infants, however there remains a significant risk for long term sequelae [2]. Adverse outcomes include an increased risk of cerebral palsy, sensory impairments, respiratory problems, impaired motor function, cognitive and attention deficits, social-emotional problems and psychiatric disorders, compared with birth at term (37–42 weeks’ gestation) [3, 4]. These problems are inversely associated with gestational age at birth and can persist from infancy into adulthood [3].

Across Europe many studies have investigated survival and long term outcomes following VPT birth, generating important data for healthcare policy and planning. However, given the relatively small numbers of infants born VPT, individual cohort studies often have insufficient sample sizes for studying rare outcomes, and are time and place specific, with differing health, education and social welfare systems across countries. These limit the generalisability of individual study findings.

The RECAP Preterm (Research on European Children and Adults born Preterm) project brings together data from VPT and very low birth weight (VLBW; < 1500 g) cohort studies across Europe to accumulate sample sizes with adequate power to better understand the health, development and quality of life of individuals born VPT. A sustainable web-based interactive platform was developed (https://platform.recap-preterm.eu/) to incorporate data from 23 birth cohort studies from 14 European countries: UK, Ireland, France, Italy, Germany, Netherlands, Portugal, Belgium, Poland, Finland, Norway, Denmark, Sweden and Estonia. Data collection for these cohorts as a whole covers a 42-year period (from 1978 onwards), spanning pregnancy, birth, and neonatal care with extensive follow-up throughout childhood and, in some studies, up to 30 years of age. Data collection methods vary between studies but predominantly comprise parent, teacher or self-report interviews or questionnaires, clinical and medical assessments and standardised psychometric tests.

The platform comprises a suite of web-based applications based on the OBiBa (https://www.obiba.org/) open-source software for epidemiological data management, analysis and dissemination, customised for use in the RECAP Preterm project. This software allows the integration of a classification system within the search function of the platform allowing variables on a topic of interest to be identified and organised to facilitate data harmonisation and analyses aiding the findability of data in line with the FAIR data principles (https://www.go-fair.org/fair-principles/). Existing data schemas and classification systems [5–7] did not cover the range and detail of the topic areas relevant for VPT birth cohort studies or were too complex in structure to organise cohort data and to implement within a search function on the RECAP Preterm platform. Therefore, we developed a bespoke classification system for VPT birth cohort studies.

This paper describes the development and use of the RECAP Preterm classification system as a fundamental component of the RECAP Preterm platform to aid data harmonisation and inform data collections for future VPT birth cohort studies.

## Method

### Participants and methodology

**Fig. 1:**
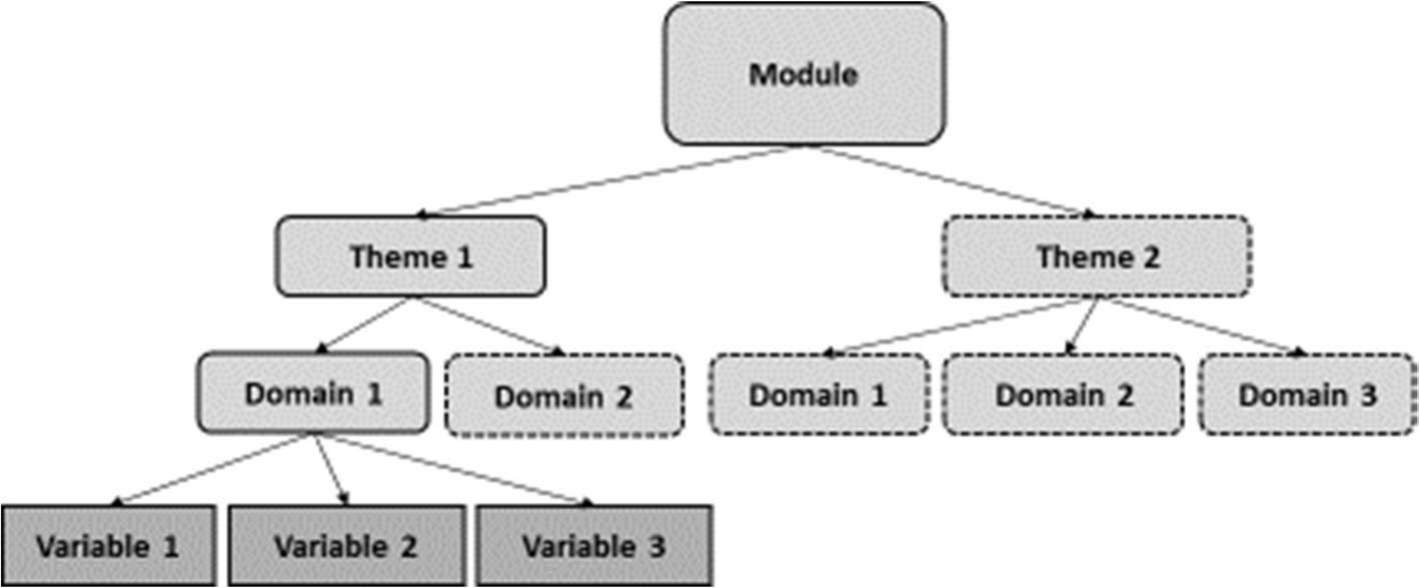
Hierarchical tree structure of the RECAP Preterm classification system showing Module (top level), Themes (second level), Domains (third level) and three variables mapped to Domain 1

The starting point for the classification system was establishing an initial set of topic areas that we expected to be common across VPT birth cohort studies. To do this, we firstly convened a multidisciplinary working group of individuals from the University of Leicester (UK) with expertise in perinatal epidemiology, neonatology, paediatrics, developmental psychology, and the management and analysis of cohort study and routine data. The group identified topics relating to the pregnancy, perinatal and neonatal periods, socio-demographic characteristics, health and neurodevelopmental outcomes. We then scoped the literature to refine these topics and identify new topics relating to additional child and adult outcomes such as education and learning, healthcare utilisation, and emotion, behaviour and mental health. We checked terms used within the classification system against reference terminologies where appropriate [8, 9]. We organised these topics into a hierarchical tree framework (Fig. 1), as used by other data harmonisation studies [10], comprising modules (top level), themes (second level) and domains (third level), to which individual cohort data variables will be mapped (fourth level). This first draft of the classification system contained 10 modules, 48 themes and 197 domains (Table 1).

**Table 1:** RECAP Preterm draft classification system

Draft Modules	
Pregnancy, Birth & Neonatal	
Socioeconomic & Demographic	
Health	
Neurodevelopment	
Education & Learning	
Emotion, Behaviour & Mental Health	
Biomarkers & Laboratory Analyses	
Social & Lifestyle	
Healthcare Utilisation & Quality of Life	
Mortality & End of Life	

**Fig. 2:**
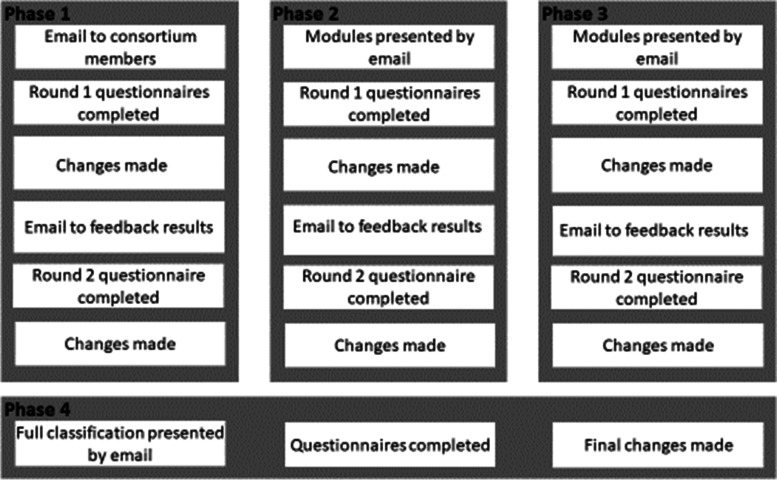
Structure of the consultation process used to assess the appropriateness of the RECAP Preterm classification system to VPT cohort data

We then consulted with experts in the study of preterm birth in a 4-phase consensus process (Fig. 2) to assess the extent to which they agreed with how the topics were organised. In Phase 1, we contacted by email all 39 members of the RECAP Preterm project and invited members who were cohort study investigators, lead clinicians and senior researchers to participate in the consensus process to assess the appropriateness of the classification system to their own cohort data. They were asked to review the whole draft classification system and two modules with accompanying themes, domains and domain definitions, and rate their appropriateness using an electronic questionnaire. Members who wished to participate had three-weeks to review the material and return completed questionnaires. We amended the classification system according to feedback received and the revised modules were circulated to all project members as a second round of consultation using the same electronic questionnaire to obtain feedback. For Phases 2 and 3, we emailed multiple modules at a time to RECAP Preterm members and again invited those who were cohort study investigators, lead clinicians and senior researchers to participate, such that each module underwent two rounds of consultation. Alterations and refinements to the contents of modules, themes, domains and domain definitions were made after each round and anonymised results were disseminated to all RECAP Preterm members at the start of each round. The final phase (Phase 4) involved obtaining feedback on the full revised classification system before making any final changes.

The final step was to map individual cohort data variables to the classification system.

### Measures

We developed an electronic questionnaire (Supplementary file 1) for the consultation with experts using ten-point Likert scales to: 1. Assess member’s level of agreement with the topic content of the classification system and each module in turn. 2. The extent to which member’s own cohort data fitted the classification system or proposed module. 3. The extent to which members agreed that the classification system or the proposed module contained relevant (modules) themes and domains. Free text comment boxes collected feedback about any changes to the content of the classification system and any proposals for the inclusion or exclusion of topics.

### Analyses

Median, minimum and maximum scores were calculated at the end of each round of consultation providing pre- and post-consultation ratings for each module to assess levels of agreement. Free text comments were summarised and categorised into overarching issues, questions and suggestions for inclusions, exclusions and amendments. These comments guided the amendments made to each module, theme, domain and domain definition at the end of each round.

## Results

Twenty-three RECAP Preterm project members, representing 12 academic or research institutions, participated in the development of the classification system, with 9 to 12 members in each round depending on their topic interest and expertise. These members were neonatologists, paediatricians, child and adolescent psychiatrists, medical physicians, physiotherapists, developmental psychologists, epidemiologists or researchers.

Table 2 shows that levels of agreement with the classification system were already high pre-consultation with a median score of eight out of 10 (minimum four; maximum 10) and this increased post-consultation to nine (seven; nine). There was an increase in levels of agreement that the classification system contained topic relevant modules, themes and domains with median scores of eight (six; nine) pre-consultation and nine (seven; 10) post-consultation. There was a reduction in the median scores for the extent to which participants’ agreed their cohort data fit the classification system, from eight (four; nine) pre-consultation to seven (four; 10) post-consultation.

**Table 2:**
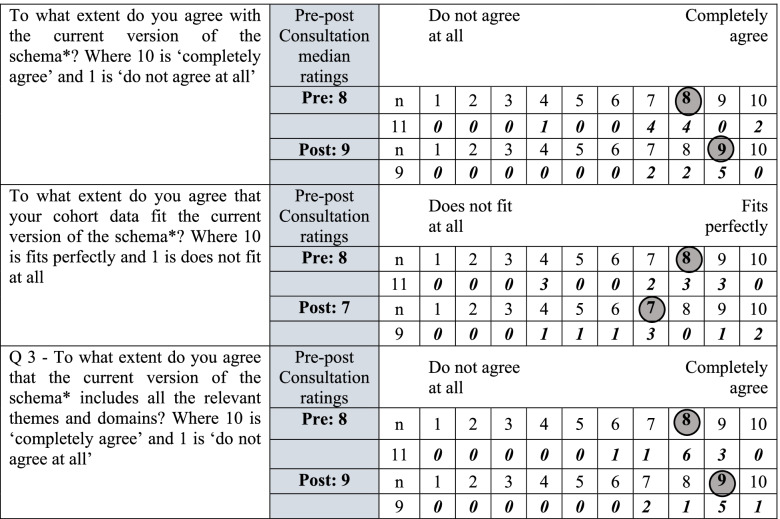
Participant’s levels of agreement with the RECAP Preterm classification system, pre- and post-consultation: circles represent the median observed value.

*‘schema’ denotes RECAP Preterm classification system

Pre-consultation, the draft classification system comprised 10 modules, 48 themes and 197 domains and following the consultation, it comprised 14 modules (Table 3), 93 themes and 345 domains.

**Table 3:** RECAP Preterm classification system: modules pre-post expert consultation

Modules pre-Consultation	Modules post-Consultation
Pregnancy, Birth & Neonatal	Antenatal & Birth
Socioeconomic & Demographic	Socioeconomic & Demographic
Health	Physical Health
Neurodevelopment	Neurodevelopment
Education & Learning	Education & Learning
Emotion, Behaviour & Mental Health	Mental Health
Biomarkers & Laboratory Analyses	Biomarkers & Laboratory Analyses
Social & Lifestyle	Social, Lifestyle & Leisure
Healthcare Utilisation & Quality of Life	Healthcare Utilisation
Mortality & End of Life	Mortality & End of Life
	Neonatal Care
	Organisational Level Information
	Health Related Quality of Life
	Administrative Information & Identifiers

The free text comments helped refine and amend the classification system so modules, themes or domains were added, removed or combined or where a concept was considered to be of higher importance they were promoted to either a domain or theme. For example, ‘Quality of Life’, which was originally part of the module ‘Healthcare Utilisation & Quality of Life’ became a module by itself (‘Health Related Quality of Life’) and new modules were created to capture ‘Organisational Level Information’ and ‘Administrative Information and Identifiers’. The original module of ‘Pregnancy, Birth and Neonatal’ became two separate modules of ‘Antenatal and Birth’ and ‘Neonatal Care’. ‘Hearing Impairments’ for example was promoted from ‘Sensory Morbidity and Treatment’ to a new theme in the ‘Neonatal Care’ module. Modules were also demoted to themes (‘Leisure Activities’ demoted from module to theme within ‘Social, Lifestyle & Leisure’ for example) or themes to domains where a concept was considered to fit within the scope of an existing module topic (for example ‘Infection’ moved from theme to domain within a combined ‘Infection & Immunity’ Theme). Names or definitions were also clarified based on feedback to help users better understand where data items should be classified.

Participants also suggested that it would be useful to organise or search for additional variable-level information relating to:

- Target – to whom the data relates
- Source – who collected the data, or who provided the data
- Mode - how the data were collected (questionnaire, interview or register/routine data)
- Instrument – the instrument used to collect the data.

**Fig. 3:**
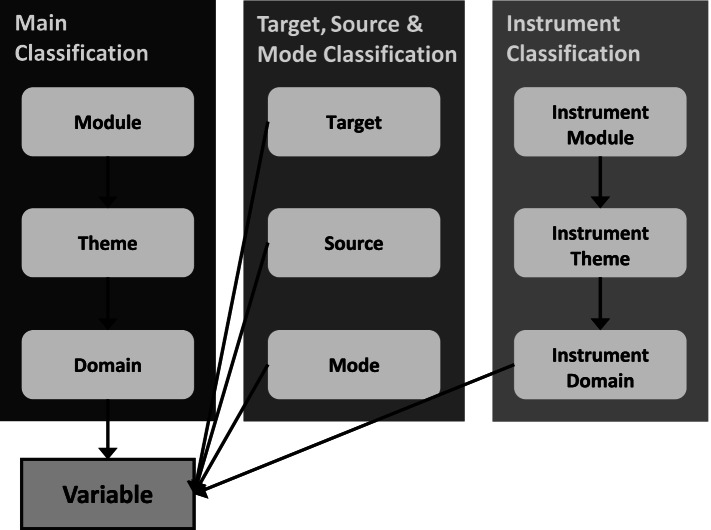
RECAP Preterm classification system structure with supplementary classifications for target, source, mode and instrument

This led to the development of supplementary classifications (Fig. 3).

For the Instrument classification, we reviewed cohort study protocols and questionnaires and found 255 versions of 188 standard instruments that had been used to collect data. These instruments formed the basis of the Instrument classification comprising seven modules (Table 4). We then liaised with RECAP Preterm project members to help identify any instruments that were incorrectly categorised or were missing from the list.

**Table 4:** RECAP Preterm classification system: Instrument Modules

Instrument Modules	
Education	
Health	
Health Behaviours	
Mental Health	
Neurodevelopment	
Quality of Life	
Social	

**Fig. 4:**
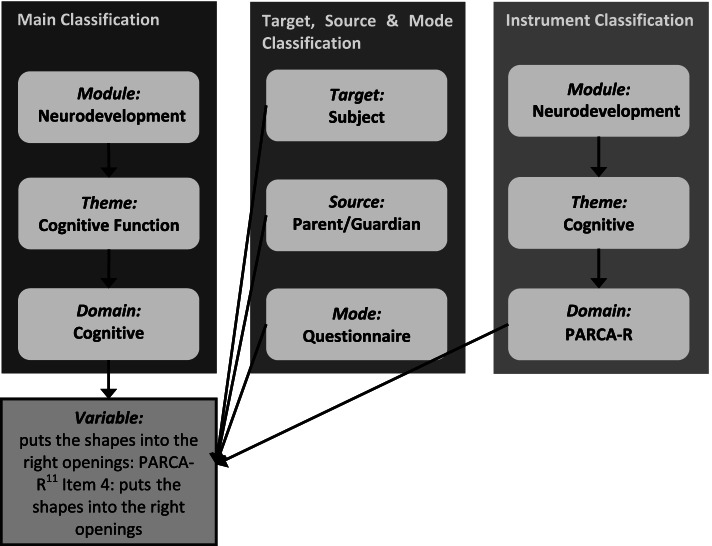
Example of a mapped data variable using the RECAP Preterm classification system

To date, we have mapped over 22,000 individual data variables to the classification system. These variables were provided by investigators of the VPT birth cohorts who participated in the RECAP Preterm project. They are now available to view and searchable within the RECAP Preterm data platform (https://platform.recap-preterm.eu). Figure 4 gives an example of how an individual cohort study variable has been mapped to the classification system.

The completed classification system can be found within the RECAP Preterm platform search function (https://platform.recap-preterm.eu/pub/search) and is also presented in full in the RECAP Preterm wiki (https://gitlab.inesctec.pt/wp4-recap/wp3/-/wikis/schema).

## Discussion

A preterm birth classification system was developed as an essential component of the RECAP Preterm project to facilitate data harmonisations to understand the long-term impact of VPT birth. It comprises topic-specific modules, themes and domains, and captures variable-level information relating to target, source, mode of data collection and the instrument used. Individual variables have been mapped to the classification system enabling platform users to identify and assess (by looking at summary statistics) similar data collected across studies in preparation for data harmonisation, and search for both raw and harmonised variables within a module, theme or domain and by target, source, mode of data collection and instrument. We developed a customised system, using methodology that has been used previously to create project-specific data schemas [10], and it has successfully been integrated within the freely accessible web-based RECAP Preterm platform. To date, over 22,000 individual cohort data variables have been mapped to the classification system and are available for use.

This classification system has several strengths. Fundamentally, it was developed in consultation with a multidisciplinary consortium of experts interested in the long-term consequences of VPT birth. Expert’s levels of agreement with the classification system and its component parts improved from an average score of eight/10 pre-consultation to nine/10 post-consultation. There was a small reduction in the pre- to post- consultation average score, from eight to seven, for the extent to which existing cohort data fit the classification system. However, the free text comments provided by the expert collaborators indicated that lower scores were given by those who only collected data up to early childhood and therefore their existing data did not fit modules that applied to outcomes in later childhood, adolescence or adulthood, for example the ‘Socioeconomic & Demographic’, ‘Education & Learning’, and ‘Health Related Quality of Life’ modules. However, this may have been expected. Our aim was to develop a classification system that would encompass the lifespan of an individual born VPT for which we used a theory-driven ‘top-down’ approach to identify relevant theoretical constructs relating to the outcomes of VPT birth. As a result, we acknowledged that there would be modules that were not directly relevant to cohorts in which data were collected up to early childhood only. However, the benefit of developing a more comprehensive classification system allows for the inclusion of further waves of data collection by existing RECAP Preterm cohorts and the integration of additional cohort studies into the RECAP Preterm platform.

Additionally, despite the RECAP Preterm project having a European focus including studies largely from high-income countries, the theoretical top-down approach we used to develop the classification system and inclusion of additional concepts aimed at future-proofing mean that it should be widely applicable allowing the integration of non-European VPT birth cohorts within the platform.

Not only did the expert collaborators provide levels of agreement with the whole classification system and its component parts, they also provided guidance as to the inclusion and exclusion of topics and the relocation of topics as modules, themes or domains based on their expert knowledge and its appropriateness to existing cohort data. The expansion of the classification system to enable data variables to be mapped to target, source, mode of data collection and instrument were also in response to expert advice. The development of the classification system was an iterative process and any conflicts in the guidance provided were considered by two members of the research team in consultation with topic experts where required.

The classification system also has limitations. The cohort studies that currently make up the RECAP Preterm project include participants aged up to around 30 years of age so information on VPT birth and aging or geriatric measures or instruments are not included. However, where possible we have future-proofed the classification system so that its structure lends itself to the inclusion of additional topics.

## Conclusion

The RECAP Preterm data classification system is a comprehensive structure comprising 14 modules, 92 themes, 345 domains to which over 22,000 cohort data variables have been mapped. Supplementary classifications capture additional information relating to target, source, mode and instrument at the variable level. It continues to develop as retrospective data items are included and mapped, as harmonised variables are created and as new data are shared. In addition to facilitating sharing of existing data, it can also be used to inform the development of future studies and data collection waves for existing cohorts. It is freely available for use worldwide to facilitate research to understand and improve lifespan outcomes for children and adults born VPT.

## Supplementary Information

**Additional file 1.**

## Data Availability

the datasets used/or analysed during the current study are available from the corresponding author on reasonable request.

## Abbreviations

RECAP Preterm: Research on European Children and Adults Born Preterm project
VPT: Very preterm
VLBW: Very low birth weight
