# Supplementary material for: Development of a data classification system for preterm birth cohort studies: the RECAP Preterm project
Source: BMC Med Res Methodol. 2022 Jan 7;22:8. doi: 10.1186/s12874-021-01494-5 (PMC8742427; doi:10.1186/s12874-021-01494-5)
Supplement: Supplementary file 1 — Additional file 1. [file 12874_2021_1494_MOESM1_ESM.docx]

Supplementary file 1: RECAP Preterm classification system consultation questionnaire

1. To what extent do you agree with the overall structure of the proposed schema?

Please mark on the scale below where 10 is ‘completely agree’ and 1 is ‘do not agree at all’.

Do not agree at all Completely agree

1 2 3 4 5 6 7 8 9 10

□ □ □ □ □ □ □ □ □ □

1. To what extent do you agree that your cohort data fit the overall structure of the proposed schema?

Please mark on the scale below where 10 is ‘fits perfectly’ and 1 is ‘does not fit at all’.

Does not fit at all Fits perfectly

1 2 3 4 5 6 7 8 9 10

□ □ □ □ □ □ □ □ □ □

1. To what extent do you agree the overall structure of the proposed schema includes all the relevant modules, themes and domains?

Please mark on the scale below where 10 is ‘completely agree’ and 1 is ‘do not agree at all’.

Do not agree at all Completely agree

1 2 3 4 5 6 7 8 9 10

□ □ □ □ □ □ □ □ □ □

3a. Is there anything you would change in the proposed schema?

No □

Yes □

If yes, please explain:

3b. Is there anything you would add to the proposed schema?

No □

Yes □

If yes, please explain:

3c. Is there anything you would remove from the proposed schema?

No □

Yes □

If yes, please explain:

THANK YOU FOR TAKING THE TIME TO GIVE US YOUR OPINION ON THIS MODULE.

1. To what extent do you agree with the proposed structure of the module?

Please mark on the scale below where 10 is ‘completely agree’ and 1 is ‘do not agree at all’.

Do not agree at all Completely agree

1 2 3 4 5 6 7 8 9 10

□ □ □ □ □ □ □ □ □ □

1. To what extent do you agree that your cohort data fit the module?

Please mark on the scale below where 10 is ‘fits perfectly’ and 1 is ‘does not fit at all’.

Does not fit at all Fits perfectly

1 2 3 4 5 6 7 8 9 10

□ □ □ □ □ □ □ □ □ □

1. To what extent do you agree that the proposed structure of the module includes all the relevant themes and domains?

Please mark on the scale below where 10 is ‘completely agree’ and 1 is ‘do not agree at all’.

Do not agree at all Completely agree

1 2 3 4 5 6 7 8 9 10

□ □ □ □ □ □ □ □ □ □

3a. Is there anything you would change in the module?

No □

Yes □

If yes, please explain:

3b. Is there anything you would add to the module?

No □

Yes □

If yes, please explain:

3c. Is there anything you would remove from the module?

No □

Yes □

If yes, please explain:

THANK YOU FOR TAKING THE TIME TO GIVE US YOUR OPINION ON THIS MODULE.
